# Mental and physical health profile of Syrian resettled refugees

**DOI:** 10.1017/S146342362200007X

**Published:** 2022-03-28

**Authors:** Rahel S. Bosson, Monnica T. Williams, Victoria A. Powers, Ruth M. Carrico, Virginia Frazier, Julio A. Ramirez, Wei Shuang Schneider, Lisa M. Hooper

**Affiliations:** 1 Global Health Center, Division of Infectious Diseases, School of Medicine, University of Louisville, Louisville, KY, USA; 2 School of Psychology, University of Ottawa, Ottawa, ON, Canada; 3 Department of Psychology, University of Miami, Coral Gables, FL, USA; 4 Spalding University, School of Professional Psychology, Louisville, KY, USA; 5 Center for Educational Transformation, University of Northern Iowa, Cedar Falls, IA, USA

**Keywords:** domestic health screen, mental health, refugees

## Abstract

**Background:**

Newly arriving Syrian refugees can present with specific health characteristics and medical conditions when entering the United States. Given the lack of epidemiological data available for the refugee populations, our study examined the demographic features of Syrian refugees resettled in the state of Kentucky. Specifically, we examined mental and physical health clinical data in both pre-departure health screenings and domestic Refugee Health Assessments (RHA; Kentucky Office for Refugees, n.d.) performed after resettlement.

**Method:**

The current study adopted a cross-sectional research design. We analyzed outcome data collected from participants from 2013 and 2015. Specifically, a comparative cross-sectional analysis was performed using clinical data from Syrian refugees who underwent an RHA as part of the resettlement process between January 2015 and August 2016. Those data were compared to data derived from refugees from other countries who resettled in Kentucky between 2013 and 2015.

**Results:**

Mental health screenings using the Refugee Health Screener (RHS-15; Hollifield *et al*., 2013) found that 19.5% (*n* = 34) of adult Syrian refugees reported signs and symptoms from posttraumatic stress, depressive symptoms, and/or anxiety, and nearly 40% (*n* = 69) reported personal experiences of imprisonment or violence, and/or having witnessed someone experiencing torture or violence. Intestinal parasites and lack of immunity to varicella were the most prevalent communicable diseases among Syrian refugees. Dental abnormalities and decreased visual acuity account for the first and second most prevalent non-communicable conditions. When comparing these results to all refugees arriving during the same years, significant differences arose in demographic variables, social history, communicable diseases, and non-communicable diseases.

**Conclusion:**

This study provides an initial health profile of Syrian refugees resettling in Kentucky, which reflects mental health as a major healthcare concern. Posttraumatic stress and related symptoms are severe mental health conditions among Syrian refugees above and beyond other severe physical problems.

## Introduction

During 2015, Kentucky admitted more than 2,000 refugees from Bhutan, Burma, the Democratic Republic of Congo, Somalia, Iran, and Iraq and welcomed more than 1,000 Cuban-Haitian entrants into the state, making it the 12^th^ largest resettlement site in the United States (Office of Refugee Resettlement [ORR], 2016). When refugees arrive into their new communities, healthcare utilization is emphasized.

To enhance optimal health in the new environment, the accurate identification of health risks and health issues is critical. To that end, newly arriving refugees are provided an opportunity to receive a review of overseas medical and socio-ethnographic history, age-appropriate immunizations, a health assessment, and laboratory screenings known as a domestic Refugee Health Assessments (RHA; Kentucky Office for Refugees, n.d.). The health screening encompasses three primary aims: (a) identify existing health conditions; (b) provide appropriate interventions for critical health needs; and (c) serve as a bridge from a palliative care model to a primary care model (Grudzen *et al.*, 2011). The health screening data are compiled into an annual report outlining the state of health among newly arriving refugees settled in Kentucky (eg University of Louisville Global Health Center, 2016). These reports become a basis for the training of healthcare providers and their teams and serve as a method of describing and recognizing specific and varied health issues among refugee populations, as well as ensuring a culturally tailored approach to their care (eg University of Louisville Global Health Center, 2016).

The current study provides an overview of the crisis leading to the resettlement of Syrian refugees in Kentucky during 2015–2016. In addition, it provides critical information evidenced during their RHAs. Conclusions and recommendations are discussed regarding the presenting mental health and medical care issues of the Syrian refugees as they transition into their new Kentucky communities. These data can help inform preventive care efforts and healthcare policy.

### Syrian crisis

In March of 2011, protests against the Syrian regime marked the starting point of a crisis in Syria (United Nations High Commissioner for Refugees [UNHCR], 2020). According to the UNHCR (2020), over six million Syrians have fled the country and reside in Turkey, Lebanon, Jordan, Egypt, and Iraq. In addition, more than six million are internally displaced. Approximately 10% live in refugee camps, and over 60% of Syrian refugees live in poverty (UNHCR, 2020). Importantly, only a few years prior to 2016, Syria was a major host country for refugees, although by 2016, Syria led the world in refugees fleeing the region (Quosh *et al*., 2013).

In light of the sheer number of refugees fleeing Syria, countries around the world have struggled with how to safely and effectively address this crisis. Among others, issues such as access to appropriate healthcare, malnutrition, and social marginalization are all vital factors related to the daily experience of displaced persons (Mowafi, 2011). Therefore, as refugees arrive at the United States, it is vital to understand their specific healthcare needs in order to adequately address them and decrease the likelihood of chronic illness and the exacerbation of mental health symptomatology. The results of the current study provide relevant epidemiological data to produce culturally tailored population-specific guidelines and to facilitate the effective utilization of healthcare services provided to resettled refugees.

### Domestic RHA

Prior to arrival into the United States, the process of resettlement starts with specific pre-departure procedures including medical screening for (a) Exclusionary purposes: Class A and Class B conditions; and (b) Immunizations. Overseas medical screenings take place 6 months before departure, to identify acute and chronic conditions that may affect the refugee or the host country. Class A conditions are conditions that may prevent refugees from entering the United States and include diseases of public health significance (eg serious mental health disorders, untreated syphilis, or active Hansen’s disease). Class B conditions are those that may interfere with the well-being of the refugee in the United States but do not preclude entry (eg latent tuberculosis (TB), HIV, diabetes, and hypertension). Approximately 24–48 h before departure, all refugees are given a final physical examination before being cleared for resettlement.

Once in the United States, the receiving communities will again provide a health screening. The Centers for Disease Control and Prevention (CDC) has provided guidelines for the performance of refugee domestic RHAs (CDC, 2012a; 2012b). These guidelines address identification of communicable and non-communicable health conditions at the time of arrival with an emphasis on preventive intervention and chronic disease management (CDC, 2012a; 2012b). The RHA is typically administered within 90 days of resettlement. The screen includes verification of demographic information, review of overseas medical information, a social and ethnographic history, medical history, mental health screening, laboratory testing including parasite and TB screening, physical exam, and referrals (University of Louisville Global Health Center, 2016). Each state follows a standardized method of employing the RHA. For example, in Kentucky, there are at least five sites providing RHAs for refugees and they all use a standard data collection tool, which permits the sites to submit electronic or paper copies to the research coordination center at the University of Louisville Global Health Center.

Given the scope of the Syrian crisis, we were interested in the extent to which Syrian refugees present with specific challenges different than other refugees. Thus, the purpose of the current study was to characterize the general epidemiological and clinical features of our target population, Syrian refugees, compared to a general population of relocated refugees. We used four sites (see below) and a data analytic-descriptive approach to explore the prevalence of specific health outcomes.

## Method

### Sample

In 2015–2016, approximately 174 Syrian refugees resettled in Kentucky. More than half of these individuals identified as male, married, and arriving from Jordan. As required, all 174 refugees completed an RHA. Complete details are provided in Table 1.

**Table 1.** Demographic characteristics of Syrian refugees

Demographic characteristic	*n*	%
Female	75	43
Male	99	57
Age (years)		
<18	101	58
18–24	11	6
25–34	32	19
35–44	19	11
45–64	11	6
65+	0	0
Marital status – adult *n* (73)		
Married	52	71
Separated	1	1
Single	14	19
Widowed	2	3
Unscreened	4	6
Country of departure		
Jordan	129	74
Turkey	40	23
Egypt	5	3
Immigration status		
Refugee	172	98
Asylee	1	1
Missing	2	1
Class B TB		
No	169	97
Yes	1	1
Unknown/missing	4	2
Class B other medical conditions		
No	122	70
Yes	49	28
Unknown/missing	3	2

### Data collection procedure

RHAs for Syrian refugees were conducted in four designated sites in Kentucky. Upon completion of the RHA collection forms, participant data were sent to the research Lab at the University of Louisville. The data were then entered into the refugee health database, which is housed in the Research Electronic Data Capture System (REDCap), a secured, password-protected system (Harris *et al*., 2009).

### Measures

The RHA used in Kentucky consisted of approximately 800 data elements and included a concomitant data dictionary to ensure consistency across all health screening sites. The mental health screening was conducted using the RHS-15 screening tool (Hollifield *et al.*, 2013), which is a 15-item measure designed to detect emotional distress in refugees who have resettled, including 13 symptom items, one item regarding coping, and a distress thermometer. Refugees respond to the RHS-15 items using a Likert-type scale (0 = not at all, 4 = extremely) allowing refugees to endorse the level of difficulty they experience from each of the symptoms presented. A positive score is identified by a score of 12 or more and/or a score greater than five on the distress thermometer. This scale has demonstrated good specificity and sensitivity in detecting mental health concerns (Hollifield *et al.*, 2013). To ensure internal consistency, researchers performed routine reliability and validity analyses, which were documented in the University’s annual report and for use in ad hoc analysis regarding the refugee health program. The domestic health data from four screening sites in Kentucky were aggregated, and analysis was performed.

### Study design and data analytic procedures

The current study was conducted following the approval of the university’s Institutional Review Board. This study adopted a cross-sectional research design. We calculated descriptive statistics and analyzed outcome data collected from participants (ie the resettled Syrian population in Kentucky) from 2013 and 2015. Specifically, a comparative cross-sectional analysis was performed using clinical data from Syrian refugees who underwent an RHA as part of the resettlement process between January 2015 and August 2016. The measurement outcomes were then used to compare to refugees who were not Syrian and who had resettled (ie arrived) in Kentucky between 2013 and 2015.

Data analytic procedures were performed using SPSS 25.0 software (IBM, NY). We used chi-square tests to measure whether the characteristics of the study variables were different between the 174 Syrian refugees and the 6040 refugees who were not Syrian. The chi-square test results indicate whether the difference between positive and negative rates of a variable is significantly different between groups (eg comparing the rate of positive and negative latent TB for Syrians and non-Syrians). Fisher’s exact test was used to maintain test precision when the count per condition was less than 5 (see Table 2).

**Table 2.** Comparison of all refugees (2013–2015) to Syrian refugees (2015–2016)

Variable	Descriptive Statistics of all refugees resettled in State of Kentucky between 2013–2015 (%)	Descriptive Statistics of Syrian refugees resettled in State of Kentucky between 2015–2016 (%)	χ^2^ value (df), significance
*n*	6040	174	–
Age, median (IQR)	27.2 (25.1)	14.5 (25)	–
Marital status (Age ≥18, *n* (%)			
Married	1971 (32.6)	52 (29.9)	.581 (1), .446
Divorced	169 (2.8)	0 (0.0)	Fisher’s exact, *P* = .02^*^
Widowed	103 (1.7)	4 (2.3)	Fisher’s exact, *P* = .55
Separated	35 (0.6)	1 (0.6)	Fisher’s exact, *P* = .99
Single	1644 (2.7)	14 (8.0)	31.78 (1), *P* < .001^*^
Single living with a partner	88 (1.5)	0 (0.0)	Fisher’s exact, *P* = .180
Social history			
Tobacco use, *n* (%)	571 (9.5)	14 (8.0)	.393 (1), *P* = .531
Alcohol abuse (Age ≥18), *n* (%)	558 (9.2)	1 (0.6)	Fisher’s exact, *P* < .001^*^
Witnessed or Experienced Torture, *n* (%)	1138 (18.8)	69 (39.7)	46.82 (1), *P* < .001^*^
RHS-15 Positive Screen, *n* (%)	946 (15.7)	34 (19.5)	1.92 (1), *P* = .166
Communicable diseases			
Latent TB	158 (2.6)	13 (7.5)	14.90 (1), *P* < .001^*^
HIV Disease, *n* (%)	40(0.7)	0 (0.0)	Fisher’s exact
Chronic Hepatitis B (HbSAg Positive), *n* (%)	120 (2.0)	2 (1.1)	Fisher’s exact
Intestinal Parasites, *n* (%)	1063 (17.6)	27 (15.5)	.507 (1), *P* = .476
Immunity to Varicella (Age ≥18)	3179 (52.6)	18 (10.3)	121.08 (1), *P* < .001^*^
Non-communicable disease			
Diabetes, *n* (%)	134 (2.2)	3 (1.7)	Fisher’s exact, *P* = 1.00
Hypertension, *n* (%)	574 (9.5)	6 (3.4)	7.33 (1), *P* = .007^*^
Decreased Visual Acuity, *n* (%)	858 (14.2)	33 (19)	3.12 (1), *P* = .07
Obesity (BMI ≥ 30 kg/m^2^, Age ≥18), *n* (%)	842 (13.9)	19 (10.9)	1.29 (1), *P* = .26
Anemia, *n* (%)	471 (7.8)	28 (16.1)	15.75 (1), *P* < .001^*^
Hyperlipidemia, *n* (%)	496 (8.2)	9 (5.2)	2.09 (1), *P* = .148
Dental Abnormalities, *n* (%)	1257 (20.8)	112 (64.4)	186.80 (1), *P* < .001^*^
Pediatric Blood Lead Level ≥5 ug/dL, *n* (%)	93 (1.5)	1 (0.6)	Fisher’s exact, *P* = .524
Myalgia/Arthralgia, *n* (%)	403 (6.7)	16 (9.2)	1.71 (1), *P* = .19
Abnormal Comprehensive Metabolic Panel, *n* (%)	766 (12.7)	18 (10.3)	.876 (1), *P* =.35

*Note*.

* Denotes a significant test value.

## Results

The majority of screened Syrian refugees were males, at 57% (*n* = 99). Ages ranged from 1 year to 64 years old, with the majority (58%, *n* = 101) below age 18 (see Table 1). Seventy-four percent (*n* = 129) of resettled Syrian refugees departed from Jordan. Ninety eight percent (*n* = 172) entered the United States under refugee status, with approximately 1% (*n* = 1) classified only as asylees. Overseas medical records indicated approximately 1% (*n* = 1) designated Class B – TB status and 28% (*n* = 49) are granted Class B for other medical conditions (see Table 1).

### Social history

While 32.6% (*n* = 1971) of all resettled adult refugees in Kentucky (2013–2015) were married, the Syrian group showed a lower percentage – although not significant – of married individuals 29.9% (*n* = 52). Interestingly, while only 2.8% of all refugees were divorced, 0% of Syrians were (Fisher’s exact test, *P* = .02). Further, more Syrians were single than all refugees (8% versus 2.7%, χ^2^ = 31.78 (1), *P* < .001).

Tobacco use was just as prevalent among adult Syrian refugees (*P* = 8.0%) when compared to 9.5% (*n* = 571) of all resettled refugees. It should be noted that 41% (*n* = 42) of Syrian children were exposed to secondhand smoke. Alcohol abuse among adults was negligible among Syrian refugees at 0.6% (*n* = 1) when compared to 9.2% (*n* = 558) in all resettled refugees, which represents a significant difference (Fisher’s exact, *P* < .001). The RHS-15 found 19.5% (*n* = 34) positive (which is 53% of tested Syrian refugees); similarly, the RHS-15 was positive in 15.7% (*n* = 946) in all resettled refugees. Syrian refugees who reported imprisonment, torture, or witnessed someone experiencing torture accounted for 39.7% (*n* = 69) of the screened Syrian group, which is almost 20% greater than the reported rate of 18.8% (*n* = 1138) for all resettled refugees (χ^2^= 46.82, *P* < .001). This significant difference indicated that mental health is a specific area on which providers ought to focus and area that additional treatment (viz., psychiatric and mental health care) is warranted within the current Syrian refugee group (see Table 2).

### Communicable diseases

An examination of latent TB screenings showed a significant difference between 7.5% of Syrian refugees (*n* = 13) and 2.6% of all settled refugees (*n* = 158), χ^2^= 14.90 (1), *P* < .001). While Syrian refugees had no positive results from sexually transmitted diseases screenings (HIV, syphilis, gonorrhea, and chlamydia), 0.7 % (*n* = 40) tested positive for HIV disease in all resettled refugees; however, this difference is non-significant. Chronic hepatitis B (HbSAg-positive) was present in 1.1% (*n* = 2) of Syrian refugees, which is close to rates found in all resettled refugees 2.0% (*n* = 120). However, 33.3% (*n* = 58) of Syrian refugees were unvaccinated for hepatitis B and susceptible to infection. Adult Syrian refugees showed significant low immunity to varicella; only 10.3% (*n* = 18) were found to be immune, while 52.6 % (*n* = 3179) of all resettled refugees were immune to varicella, χ^2^ = 121.08 (1), *P* <.001 (see Table 2).

### Non-communicable diseases

Results revealed that 1.7% (*n* = 3) of Syrian refugees were diabetic (random blood glucose level ≥ 200 mg/d), which was similar to rates found in all resettled refugees 2.2% (*n* = 134). No evidence was found in the reviewed medical records of hypertension-established diagnoses based on multiple measurements and follow-ups. There were *possible or sub-clinical* diagnoses of hypertension (systolic >140 mmHg or diastolic > 90 mmHg) in 3.4% (*n* = 6) of adult Syrian refugees (15% of tested adults), which was significantly less than the rates (9.5%) among all resettled refugees (*n* = 574), χ^2^ = 7.33 (1), *P* = .007. Domestic RHAs provided more reliable and accurate information than overseas medical records about the prevalence of obesity among resettled Syrian refugees.

Body mass index (BMI) screenings among adults (*n* = 73) indicated 26% (*n* = 19) were overweight (BMI 25.0-29.9) and 26% (*n* = 19) were obese (BMI > 30.0), which is slightly higher, though not significantly different, than rates found in all adult resettled refugees (see Table 2). BMI percentile screenings among Syrian children (*n* = 101) denoted only 2% (*n* = 2) were either overweight or obese. Hyperlipidemia was found in 5.2% (*n* = 9) of Syrian refugees (indicated by a cholesterol level ≥ 240 mg/dL), while 8.2% in the general resettled refugee population had hyperlipidemia (*n* = 496), χ^2^ = 2.09 (1), *P* = .148; however, it accounted for 14% of the Syrian refugees whose cholesterol levels were tested.

Anemia was twice as prevalent among Syrian refugees; 16.1% (*n* = 28) of screened Syrian refugees were anemic, while anemia was present in 7.8% (*n* = 471) of all resettled refugees, χ^2^ = 15.75 (1), *P* < .001. Moreover, mean corpuscular volume (MCV) values of Syrian refugees showed that 37.4% (*n* = 65) of tested Syrian refugees had MCV below normal (<80 fL/red cell), which indicates deficiency of iron as the underlying cause of anemia. An abnormal comprehensive metabolic panel was found in 10.3% (*n* = 18) of Syrian refugees, which matches rates found among all resettled refugees 12.7% (*n* = 766). Myalgia and/or arthralgia were found in 9.2% (*n* = 16) of Syrian refugees, which are higher but non-significant rates than those among the rest of resettled refugees.

Decreased visual acuity was present in 19% (*n* = 33) of Syrian refugees, a higher but non-significant rate compared to those found among all resettled refugees, 14.2% (*n* = 858). Sixty-four percent (*n* = 112) of Syrian refugees showed dental abnormalities, which are more than three times the rates (20.8%) found in all resettled refugees (*n* = 1257), χ^2^= 186.80, *P* <.001. Screening for lead poisoning among pediatric refugees showed only 0.6 % (*n* = 1) positive results among the Syrian group, which is comparable to 1.5% (*n* = 93) of all resettled refugees who showed elevated lead levels (Table 2).

Based on reviewing doctors’ notes from the RHAs, 86.8% (*n* = 151) of Syrian refugees had at least one medical condition that required either treatment, follow-up, or referral to a specialist. The top ten diagnoses identified by RHA healthcare professionals were as follows: (1) Dental Abnormalities; (2) Abnormal Urine Analysis; (3) Mental Health Issues (PTSD, anxiety, and increased levels of stress); (4) Anemia (iron deficiency anemia); (5) Obesity; (6) Intestinal parasites; (7) Decreased Visual Acuity; (8) Myalgia, arthritis, and back pain; (9) TB exposure; and (10) Tobacco use.

## Discussion

The current cross-sectional study examined mental and physical health clinical data in both pre-departure health screenings and domestic RHAs (Kentucky Office for Refugees, n.d.) performed after resettlement. We used a comparative cross-sectional analysis to characterize the general epidemiological and clinical features of a target population compared to a general population of relocated refugees. A review of the current literature showed a lack of published data describing the current health status of Syrian refugees in resettlement countries, with the exception of a report in November 2015 by the Canadian Immigration Services (CIC, 2015), and one study published the same year regarding the prevalence and care-seeking for chronic diseases among Syrian refugees in Jordan (Doocy *et al*., 2015). Thus, there is a notable lack of relative information specifically in the United States. The current study extends previous literature regarding Syrian refugees in several ways. First, the Canadian report (CIC, 2015) explored cultural variables, without a focus on the epidemiological factors necessary to improve well-being. Second, research findings by Doocy *et al.* (2015) are based on data derived from self-report as opposed to clinical data. Additionally, the study performed in Jordan focused on non-communicable diseases only, thus limiting the generalizability of the results. Other published literature has focused on either one specific condition, such as mental health among Syrian refugees, or the humanitarian response and displacement crises facing displaced Syrians in neighboring countries.

In contrast to previous studies on Syrian refugees, the current study focused on both mental health and medical conditions and health characteristics of Syrian refugees, which are based on standardized clinical measurements and lab results. The significant higher number of Syrian refugees compared to all resettled refugees who witnessed or experienced torture indicated that the Syrian refugees require more mental and psychological care compared to all the resettled refugees. Additionally, the more severe conditions of Syrian refugees in terms of non-communicable disease require a higher demand for medical care from the local hospitals. In light of the Syrian crisis and the dearth of information on Syrian refugees, a descriptive analysis of the currently available data appeared to be the most appropriate approach. As such, the current study helps lay the foundation for more comparative research once more data are made available from other states.

### Limitations

The current study’s findings ought to be considered in the context of the study’s limitations. First, the analyses were based on pre-existing data. Consequently, other measures could not be added and other factors could not be examined. Second, although there was a substantive amount of information, the database also contained missing information, which may have resulted in a less complete description of the health status of the Syrian refugee sample. Third, the small sample size serves as a limitation of the study. Fourth, the data analyses were delimited to descriptive results. Under the condition where follow-up information of the Syrian refugees is collected, predictive models could be used to investigate how the underlying medical conditions may influence their quality of life. Given the small sample of Syrian refugees in the current study, it remains less clear if the current study’s findings would be evinced among

## Conclusion

Syria has been identified as an important origin country from which the highest number of refugees are derived (UNHCR, 2020). Newly arriving Syrian refugees can present with specific health characteristics and medical conditions when entering the United States. Given the high number of Syrian refugees, it is critical to understand their health status and to establish what behaviors, risks, and potential buffers are implicated in short- and long-term health outcomes. Toward this end, this study provides an important health profile of Syrian refugees resettling in Kentucky and finds that mental health emerged as a major concern. In addition, tobacco use, obesity, and dental issues are all prevalent issues among Syrian refugees. Consequently, Syrian refugees in the current sample appear to be more prone to specific risk factors and comorbid mental health and medical conditions than other refugees. Attention toward preventive services used by Syrian refugees represents an urgent goal of healthcare planning. Additionally, these findings can preliminarily help inform healthcare policy.
